# Psychiatric illness and regression in individuals with Phelan-McDermid syndrome

**DOI:** 10.1186/s11689-020-9309-6

**Published:** 2020-02-12

**Authors:** Teresa M. Kohlenberg, M. Pilar Trelles, Brittany McLarney, Catalina Betancur, Audrey Thurm, Alexander Kolevzon

**Affiliations:** 1 Department of Psychiatry, University of Massachusetts Medical School, Worcester, MA USA; 2 Seaver Autism Center for Research and Treatment, Icahn School of Medicine at Mount Sinai, New York, NY USA; 3 Department of Psychiatry, Icahn School of Medicine at Mount Sinai, New York, NY USA; 4 Phelan-McDermid Syndrome Foundation, Osprey, FL USA; 5 Sorbonne Université, INSERM, CNRS, Neuroscience Paris Seine, Institut de Biologie Paris Seine, Paris, France; 6 Neurodevelopmental and Behavioral Phenotyping Service, Intramural Research Program, National Institute of Mental Health, National Institutes of Health, Bethesda, MD USA

**Keywords:** Phelan-McDermid syndrome, *SHANK3*, Mania, Depression, Bipolar disorder, Psychosis, Catatonia, Regression

## Abstract

**Background:**

Phelan-McDermid syndrome (PMS) is a genetic condition characterized by intellectual disability, speech and language deficits, hypotonia, autism spectrum disorder, and epilepsy. PMS is caused by 22q13.33 deletions or mutations affecting *SHANK3*, which codes for a critical scaffolding protein in excitatory synapses. *SHANK3* variants are also known to be associated with an increased risk for regression, as well as for psychiatric disorders, including bipolar disorder and catatonia. This study aimed to further describe these phenomena in PMS and to explore any relationship between psychiatric illness and regression after early childhood.

**Methods:**

Thirty-eight people with PMS were recruited to this study through the Phelan-McDermid Syndrome Foundation based on caregiver report of distinct development of psychiatric symptoms. Caregivers completed a clinician-administered semi-structured interview focused on eliciting psychiatric symptomatology. Data from the PMS International Registry were used to confirm genetic diagnoses of participants and to provide a larger sample for comparison.

**Results:**

The mean age of the 38 participants was 24.7 years (range = 13 to 50; SD = 10.06). Females (31 of 38 cases; 82%) and sequence variants (15 of 38 cases; 39%) were over-represented in this sample, compared to base rates in the PMS International Registry. Onset of psychiatric symptoms occurred at a mean age of 15.4 years (range = 7 to 32), with presentations marked by prominent disturbances of mood. Enduring substantial loss of functional skills after onset of psychiatric changes was seen in 25 cases (66%). Symptomst indicative of catatonia occurred in 20 cases (53%). Triggers included infections, changes in hormonal status, and stressful life events.

**Conclusions:**

This study confirms that individuals with PMS are at risk of developing severe neuropsychiatric illness in adolescence or early adulthood, including bipolar disorder, catatonia, and lasting regression of skills. These findings should increase the awareness of these phenotypes and lead to earlier diagnosis and the implementation of appropriate interventions. Our findings also highlight the importance of genetic testing in the work-up of individuals with intellectual disability and acute psychiatric illness or regression. Future research is needed to clarify the prevalence and nature of psychiatric disorders and regression among larger unbiased samples of individuals with PMS.

**Electronic supplementary material:**

The online version of this article (10.1186/s11689-020-9309-6) contains supplementary material, which is available to authorized users.

## Background

Phelan-McDermid syndrome (PMS) is a neurogenetic syndrome caused by haploinsufficiency of the *SHANK3* gene due to a spectrum of anomalies in the terminal region of the long arm of chromosome 22, ranging from single-nucleotide variants to large deletions affecting multiple genes [1]. SHANK3 is a key structural protein in excitatory synapses, with several isoforms with different functions in development and in the synapse [2, 3]. Deletions or sequence variants of the *SHANK3* gene are associated with the neuropsychiatric manifestations of the syndrome and required for the diagnosis of PMS [4–7]. Individuals with PMS often present with intellectual disability (ID), features of autism spectrum disorder (ASD), hypotonia, and severely delayed or absent speech [8–10]. Genotype-phenotype studies in individuals with terminal deletions have found that the severity of speech impairment and intellectual disability increases with increasing deletion size [8, 9]. In one recent study examining phenotypic manifestations among 17 individuals with sequence variants within *SHANK3* [7], the majority developed single words, and 44% had phrase speech, in contrast to more severe impairment in individuals with deletions.

Epilepsy is reported in 17% to 70% of individuals with PMS [9, 11–14]. As individuals with PMS age, they appear to be at increased risk for bipolar disorder [15–20] and an associated risk of significant cognitive and behavioral regression [5, 6, 16, 18–20]. Indeed, *SHANK3* variants have been implicated in the risk for severe neuropsychiatric disorders, including mood and psychotic disorders [15, 16, 19, 21, 22]. Gauthier et al. (2010) identified sequence variants in the *SHANK3* gene in four individuals initially diagnosed with atypical, early-onset schizophrenia, and a history of borderline to mild intellectual disability [21]. Catatonia, a unique syndrome of motor and autonomic dysregulation associated with a variety of psychiatric and medical conditions, has also been described in PMS [7, 15, 22, 23]. Of note, catatonia in pediatric populations and in individuals with developmental disabilities often goes unrecognized [24] and poorly treated [25].

A few previous cross-sectional studies have used systematic methods to characterize the behavioral profile of individuals with PMS across the lifespan [19, 26–28]. However, the nature and course of psychiatric symptoms in PMS, and in particular their association with regression occurring much later than the early childhood regressions typical of ASD, have not been extensively documented. In this retrospective study, we collected developmental histories, behavioral profiles, and genetic findings of 38 adolescents and adults with PMS and psychiatric illness with the aim to (1) better characterize the psychiatric and developmental phenomena reported in PMS and (2) aid in early recognition and treatment optimization.

## Methods

### Participants and procedures

The study was approved by the Institutional Review Board of the PMS International Registry (PMSIR) (https://www.pmsf.org/registry/). Informed consent for participation in the Registry was obtained from the parents or legal guardians of participants, who also signed a release for inclusion in publication, and for integration of their interview data with their PMSIR data. Families were recruited by outreach through the PMS Foundation community Facebook page, and families were specifically invited to participate if the individual with PMS had experienced distinct psychiatric changes, such as mood episodes, psychosis, marked changes in sleep and energy, major loss of skills, sudden new intense obsessive-compulsive behaviors, or other neuropsychiatric difficulties, with or without regression. Families either contacted the researcher (TMK) directly in response to the Facebook message or responded after other families relayed the recruitment message on Facebook to parents with possible clinical concerns as described. In addition to the 37 English-speaking families, six families were referred through the PMS Spanish Association, and two enrolled and completed the study procedures.

Thirty-seven caregivers completed interviews on 39 participants and provided informed consent; one participant was excluded from the analysis since criteria were not met for a distinct psychiatric episode. Three families had responded to the invitation but did not complete interviews or consent after an initial contact established that they did not meet criteria for a distinct psychiatric episode. The final sample included 38 individuals from 36 families, ranging in age from 13 to 50 at the time of contact. The sample includes two sets of monozygotic twins with both twins enrolled. Caregivers interviewed were mothers in all but one case, in which the respondent was a sibling who was the legal guardian. Interviews were conducted in English (*n* = 34) or Spanish (*n* = 2), and respondents lived in the USA (*n* = 29), Australia (*n* = 4), Canada (*n* = 1), England (*n* = 1), Netherlands (*n* = 1), and Spain (*n* = 2).

### Measures

A semi-structured interview entitled the Caregiver Interview for Psychiatric Illness in Persons with ID (see Additional file 1) was developed by TMK and conducted with caregivers by child and adolescent psychiatrists (TMK for English interviews and MPT for Spanish interviews). The interview includes questions designed to elicit descriptions of the participant’s developmental history, major health challenges, the emergence and course of episodes of psychiatric illness or regression, response to pharmacologic treatment, and current level of functioning. Interviews typically lasted 90 min. All final notes were reviewed and approved by the families before inclusion in the study.

Genetic reports were obtained for all participants and reviewed by the geneticist (CB) curating results for the PMSIR. PMSIR data were used to confirm genetic results for study participants and for comparisons of prevalence of genetic variants, age, and gender. However, only 21 of 38 participants in this study had completed the PMSIR clinical and developmental questionnaires, limiting direct comparisons of clinical data between study participants and other Registry members. Nine of 38 participants were not enrolled in the Registry prior to study participation.

Comparisons between functional status and developmental milestones in study participants vs Registry data included parent report of whether key developmental skills were ever achieved on the Caregiver Interview (for the study sample) and on the Registry’s Developmental Questionnaire for a group of respondents of similar age, with Registry data excluding the participants who were enrolled in the current study.

Assessment of behavioral symptoms and labeling of psychiatric diagnoses in the context of neurodevelopmental disorders is complicated by cognitive and communicative limitations, atypical presenting features, and premorbid characteristics (e.g., echolalia, repetitive behaviors). The Diagnostic and Statistical Manual for Mental Disorders, 5th edition [29] does not comprehensively include modifications of all diagnostic criteria for differential manifestations that may present in the context of intellectual disability, and there is a dearth of psychometrically rigorous instruments validated for psychiatric evaluation in this population. Therefore, we used the Diagnostic Manual - Intellectual Disability, Second Edition (DM-ID-2) [30] to classify psychiatric episodes. The DM-ID-2 is based on the DSM, adapted to include caregiver observations of behavior and to reduce the number of symptoms required to make diagnoses where persons with intellectual disability may not be able to report experiences [31]. All cases were reviewed by a child and adolescent psychiatrist (TMK) using a checklist of DM-ID-2 criteria for each of these disorders. The symptoms reported by caregivers during acute episodes met criteria for major depressive episode, manic episode, obsessive-compulsive disorder, generalized anxiety disorder, brief psychotic disorder, schizoaffective disorder, and catatonia associated with another mental disorder (see Additional file 2). In addition, two child and adolescent psychiatrists (MPT, AK) randomly reviewed and classified eight cases to assure accuracy of diagnostic classification. Agreement on the presence of a mood disorder was 100%, agreement on whether the first mood episode was depressive or manic was 88%, and agreement on the presence of catatonia was 88%.

The word “regression” is often used to describe a wide variety of states, including transient loss of skills during psychiatric episodes with and without catatonia. For clarity, “regression” was defined in this study as a prolonged loss of previously acquired skills that either (a) began when the individual was psychiatrically well or (b) began during a psychiatric episode, with loss of skills persisting for at least 6 months beyond the resolution of the psychiatric episode.

## Results

Descriptive statistics and comparisons to participants in the PMSIR are reported in Table 1. Summaries of case histories and genetic findings are reported in Table 2. There was a strong female predominance in this sample (31 females, 7 males) whereas PMS typically affects males and females in equal proportion. There were 23 individuals with terminal deletions, with a mean size of 1.63 Mb (range 160 kb–6.41 Mb). Five deletions were secondary to a chromosomal rearrangement: a ring chromosome 22 in three individuals and an unbalanced translocation in two. Twelve individuals with terminal deletions had not had a karyotype, so the possibility of a ring chromosome 22 cannot be excluded. Fifteen individuals had pathogenic sequence variants in *SHANK3* (12 frameshift variants and 3 nonsense variants).

**Table 1:** Demographic and clinical variables in the study sample as compared to the Phelan-McDermid Syndrome International Registry (PMSIR) participants 13 or older

	This study (*n* = 38)	PMSIR (*n* = 130)	Comparison
Mean age at data collection	24.7 years ± 9.92	20.8 years ± 7.65	*t*(166) = 2.56, *p* = .011 *χ*^2^(1) = 10.21, *p* = .001
Gender			
Male	18% (7/38)	47% (61/130)	*χ*^2^(1) = 10.20, p = .001
Female	82% (31/38)	53% (69/130)	
Genetic defect ^a^			
Terminal deletion	61% (23/38)	91% (118/130)	*χ*^2^(1) = 19.79, *p* < .001
Interstitial deletion	–	2% (3/130)	
*SHANK3* sequence variant	39% (15/38)	7% (9/130)	
ASD diagnosis (ever)^d^	55% (21/38)	41% (37/91^b^)	*χ*^2^(1) = 2.105, *p* = .147
History afebrile seizure(s)	39% (15/38)	41% (37/91)	*χ*^2^(1) = .044, *p* = .834
Walked independently (ever)	100% (38/38)	81% (64/79^c^)	*χ*^2^(1) = 8.212, *p* = .004
Spoke in phrases or sentences (ever)	79% (30/38)	51% (40/79)	*χ*^2^(1) = 8.317, *p* = .004
Toileted independently “always” or “sometimes” (ever)	89% (34/38)	48% (38/79)	*χ*^2^(1) = 18.029, *p* < .001
Dressed self independently (ever)	78% (30/38)	42% (33/79)	*χ*^2^(1) = 13.26, *p* < .001
Chronic constipation	84% (32/38)	15% (14/91)	*χ*^2^(1) = 55.428, *p* = <.001
Acute urinary retention	47% (18/38)	3% (3/87)	*χ*^2^(1) = 37.091, *p* < .001

^a^Among all Registry participants (*n* = 509, excluding the study participants), there are 467 terminal deletions (92%), 10 interstitial deletions (2%) and 32 sequence variants (6%)

^b^91 participants in this age range (excluding the study participants) completed the Registry Clinical Questionnaire

^c^79 participants in this age range (excluding the study participants) completed the Registry Developmental Questionnaire

^d^Prior to the onset of neuropsychiatric illness, 42% (16/38) of participants had ASD diagnoses

**Table 2:** Case summaries

Case # Gender Age at time of study	Genetic defect	Level of functioning prior to onset psychiatric illness ^1^	Reported possible triggering stressors	Age onset psychiatric illness; psychiatric diagnoses by DM-ID-2 criteria; associated features; number of episodes	Age and areas of regression after onset psychiatric illness ^2, 3^	Years since last psychiatric episode and regression; degree of recovery in that time and skills regained
Case 1 Female 18 years 9 months	Terminal deletion [89 kb] arr[hg19] 22q13.33(51,122,483-51,211,392)x1	Phrase speech Toilet trained Dressed self Fed self Independent for hygiene Read sight words Counted to 100	None noted	Age 14 First episode depressed with new tics, fecal smearing Then, OCD, new pica, manic episode and catatonia Three episodes	Age 14 Decreased speech Decreased eye contact New onset incontinence Unable to dress self Lost all academic skills	Mood episode in past year Four years since regression Minimal recovery Occasional meaningful speech and singing Intermittent feeding self with utensil
Case 2 Female 29 years 6 months	Mosaic terminal deletion and interstitial duplication [4.4 Mb] 46,XX; arr[hg19] 22q13.2q13.31(43,675,642-46,807,366)x3[0.65], 22q13.31q13.33(46,808,071-51,197,838)x1[0.65]	Single words Toilet trained Pretend play with dolls	Program transition	Age 27 First episode depressed with catatonia Then new generalized anxiety disorder (GAD) One mood episode Ongoing GAD	Age 27 Decreased speech Incontinent Decreased independence in ADLs Decreased pretend play	Two years since mood episode Two years since regression No recovery
Case 3 Female 18 years 0 month	Terminal deletion–unbalanced translocation [207 kb] 46,XX; arr[hg19] 3p26.3(180,509-2,097,153)x3,22q13.33(50,990,475-51,197,838)x1	Spoke in full sentences Toilet trained Wrote simple text Used iPad	URI Beginning new school	Age 12 First episode OCD dx PANDAS Then depressed with new tics, tremors and posturing, Then mixed manic and depressed symptoms with new aggression, new anxiety at leaving house Four episodes (three mood)	Age 15 Decreased speech clarity Frequent urinary incontinence Decreased independence in ADLs	Mood episode in past year Three years since regression Moderate recovery Still unpredictably incontinent Speech improved, still dysfluent
Case 4 Female 22 years 3 months	Terminal deletion – ring 22 46,XX,r [22](p13q13.3).ish r [22](ARSA-)	Phrase speech Never toilet trained Dressed self with prompts Read some sight words Did puzzles	URI	Age 16 First episode depressive symptoms^5^ Then manic symptoms with hand posturing and gait disturbance Three mood episodes	Age 16 Decreased speech Decreased response to environment Decreased orientation	Mood episode in past year Six years since regression Skills continue to deteriorate
Case 5 Male 36 years 1 month	Terminal deletion [106 kb] 46,XY; arr[hg18] 22q13.33(49,474,771-49,581,309)x1	Full sentences Toilet trained Dressed self Did all self-care Independent with own routines Able to return home from day program unassisted and to be home alone safely	Program transition	Age 28 First episode depressed Then new aggression which worsened to require two hospitalizations and placement in nursing care facility Three mood episodes	Age 30 Slowly regressed over several years Lost speech Decreased continence Lost ability to dress and shower Needs help with eating Moved slowly No longer can be left alone	No episode in past 3 years Six years since regression Moderate recovery Remains largely mute and uses single words or grunts Dependent for ADLs Regained some self-care skills Improved mood Incontinent at night
Case 6 Female 28 years 11 months	Terminal deletion [54 kb] arr[hg19] 22q13.33(51,123,491-51,178,264)x1	Spoke in full sentences Toilet trained Dressed self Made snacks Read simple books Wrote simple text	Pneumonia	Age 14 First episode depressed Then mania with complete insomnia for 12 days, catatonia, neuroleptic malignant syndrome and seizures Now ECT dependent 10+ mood episodes	Age 26 Sporadic incontinence Confused Couldn’t read or write Decreased hand skills Poor memory	Mood episode in past year Two years since regression No recovery
Case 7 Female 34 years 6 months	Terminal deletion – ring 22 46,XX,r [22](q13.3) with deletion of terminal 22q	Simple repetitive speech Toilet trained Dressed self with prompts Some sight words Some computer skills	None noted	Age 30 First episode depressed Then both manic and depressed episodes 10+ mood episodes	Age 30 Decreased vocalizations Frequent incontinence Decreased independence with dressing	Mood episode in past year Four years since regression Moderate recovery Vocalizes more Remains incontinent Feeds self but needs supervision for choking Needs help with dressing
Case 8 Female 36 years 3 months	Terminal deletion [106 kb] arr[hg18] 22q13.33 (49,475,238-49,581,309)×1	Phrase speech Toilet trained Dressed self with supervision Read simple words Played with dolls	None noted	Age 11 First episode manic symptoms Then mixed mood episodes 10+ mood episodes	Age 11 Steady developmental decline from age 11 Lost almost all language Incontinent Lost use of utensils No longer dresses self No play interests	No episode in past year 25 years since regression No recovery
Case 9 Female 43 years 1 month	Terminal deletion – ring 22 [2.1 Mb] 46,XX,r [22](p13q11.2); arr[hg19] 22q13.32q13.33(49,056,457-51,179,671)x1	Spoke in full sentences Toilet trained Dressed self Independent for hygiene Active in programs	None noted	Age 32 Abrupt onset of stuporous catatonia with food phobia Then multiple manic and depressed episodes 10+ mood episodes	Age 32 Decreased speech Incontinent No longer able to dress self or bathe self	Mood episode in past year 12 years since regression Minimal recovery Decreased speech Intermittent incontinence Needs supervision for self-care
Case 10 Female 17 years 11 months	Sequence variant – Frameshift NM_033517.1:c.3424_3425delCT, p.Leu1142Valfs*153	Spoke in full sentences Toilet trained Dressed self and did most hygiene Read sight words Wrote name, familiar words, copied text	Menses Started at new school	Age 14 First had three peri-menstrual manic episodes followed by paranoia Then prolonged severe depression with catatonic features Then acute OCD with tics, posturing and choreiform movements Then mania with first seizure and catatonia, dx seronegative autoimmune encephalitis Six episodes (five mood episodes)	No lasting loss of skills	Mood episode in past year No regression
Case 11 Male 14 years 11 months	Sequence variant – Frameshift NM_033517.1:c.3679dupG, p.Ala1227Glyfs*69	Phrase speech Social and interactive Toilet trained Read sight words Typed from a model	Gastro-enteritis	Age 12 First episode manic with agitation, new pica, new incontinence, destructive behavior, mutism, and catatonia Then OCD with agitation and aggression Five+ mood episodes	Age 12 Lost most language Less social and interactive Incontinent Lost all academic skills New obsessional and repetitive behaviors	Mood episode in past year Two years since regression Minimal recovery Rare speech and interaction now Still incontinent
Case 12 Female 43 years 8 months	Sequence variant – Frameshift NM_033517.1:c.4906_4921dup16, p.Pro1641Leufs*58 dn	Spoke in full sentences Toilet trained Dressed and washed Read at 3rd grade level Math grade level through 9th grade Worked at supported job in library and in town hospital Balanced own checkbook	Menses Viral infections	Age 11 First episode mixed, with agitation, insomnia, cognitive dulling, posturing Then multiple mood episodes with catatonia 20+ mood episodes	Age 11 Gradual, with each episode lost skills, twice lost ability to walk, would regain some skills with therapies, still working and verbal to age 28; after a severe cold lost all speech, continence, self-care and many motor skills, cannot use hands (dystonic posturing at wrists)	No episode in past year 15 years since most severe regression No recovery
Case 13 Female 14 years 1 month	Terminal deletion [123 kb] 46,XX; arr[hg18] 22q13.33(49,468,254-49,591,415)x1	Spoke in full sentences Toilet trained Pulled on clothes Needed help with ADLs Read at 1st grade level Wrote and typed Used iPad	Strep infection Menses	Age 9 First episode complex tics and choreiform movements w/ high strep titers, dx PANDAS and resolved with antibiotics Then rage outbursts and peri-menstrual aggression Then depressive episode with severe anxiety and new generalized anxiety disorder 3+ mood episodes	No lasting loss of skills	No episode in past year No regression
Case 14 Male 16 years 6 months	Sequence variant – Nonsense NM_033517.1:c.5008A > T, p.Lys1670* (this individual also has a likely benign missense variant in *SHANK3*, c.3872C > T, p.Ser1291Leu)	No speech, used PECS Toilet trained Fed self Used iPad Navigated internet Liked music	None noted	Age 15 First episode manic Then monthly manic episodes Then severe aggression requiring hospitalization and residential care 20+ manic episodes	Age 15 Lost toilet training Averse to touch Lost computer skills Lost social interest	No episode in past year 18 months since regression Moderate recovery of toileting, self-feeding, some computer skills
Case 15 Female 28 years 11 months	Terminal deletion [43 kb] arr[hg18] 22q13.33(49,479,705-49,522,868)x1	Single words Never toilet-trained Could change own pull-up Dress self Drink from cup	School re-entry Possible URIs	Age 9 First episode manic Then manic spells with OCD behaviors Then depressed periods with new aggression 10+ mood episodes	Age 24 Lost ability to: Dress self Change pull-up Drink from cup	Three years since last episode Four years since regression No recovery
Case 16 Female 15 years 1 month	Sequence variant – Frameshift arr[hg19] 22q13.33(51,121,452-51,197,838)x1	Spoke in full sentences Toilet trained Dressed self Read Pretend play Wrote in cursive Karate	Menses	Age 13 First episode manic with paranoia Then depressive episode with catatonia requiring ICU care and ECT Then recurrent manic episodes Five+ mood episodes	No lasting loss of skills	Mood episode in past year No regression
Case 17 Female 13 years 10 months	Terminal deletion [4.3 Mb] arr[hg19] 22q13.31q13.33(46,928,208-51,224,252)x1 dn	Single words or short phrases Toilet trained Dressed self Fed self Rode scooter	Menses	Age 13 First episode depressed with catatonia Then mania with urinary frequency and occasional incontinence, changes in gait and posture Four mood episodes	No lasting loss of skills	Mood episode in past year No regression
Case 18 Male 20 years 10 months	Terminal deletion [76 kb] arr[hg19] 22q13.33(51,121,452-51,197,838)x1	Spoke in full sentences Toilet trained Dressed self Showered self Read at 3rd grade level Wrote sentences Used calculator Used iPhone to call/text friends	Possibly his increased awareness of deficits	Age 16 First episode mixed mood disturbance with sleepiness and irritability Then mania alternating with depression and severe aggression Three+ mood episodes	Age 16 Confused Needs constant supervision Cannot dry self Puts clothes on while still wet Decreased academic skills Urinary incontinence Lost interest in usual activities	Mood episode in past year Four years since regression No recovery
Case 19 Male 26 years 5 months	Sequence variant – Nonsense NM_033517.1:c.1572C>A, p.Cys524*	Spoke in full sentences Toilet trained Independent for ADLs and travel with school groups Reading at 3rd grade level Attended community college Competitive tennis Marathoner	School transition	Age 17 First episode manic with irritability, confusion, catatonia and new aggression Then multiple manic and depressed episodes Psychotic features occurring between mood episodes 20+ mood episodes	Age 17 Gradual, lost most speech Often incontinent Lost ability to perform ADLs Requires constant 1:1 supervision	Mood episode in past year 12 years since regression No recovery
Case 20 Female 17 years 1 month	Sequence variant – Frameshift NM_033517.1:c.4065_4066delTG, p.Val1357Glyfs*4 dn	Phrase speech Asked for needs Toilet trained Fed self	Strep infection	Age 14 First episode OCD with enuresis at night, aggression and sub-threshold manic symptoms Then similar episodes of OCD with manic symptoms Later diagnosis of autoimmune encephalitis Three+ mood and OCD episodes	Age 15 Decreased intelligibility Decreased toileting Decreased communication Decreased feeding self	Mood episode in past year Two years since regression Moderate recovery of speech clarity and toileting skills
Case 21 Female (twin of case 20) 17 years 1 month	Sequence variant – Frameshift NM_033517.1:c.4065_4066delTG, p.Val1357Glyfs*4 dn	Phrase speech Toilet trained Fed self Dressed self Read a few sight words Basic writing	Strep infection	Age 14 First episode anxiety/OCD, transient inability to speak, then developed mania Then diagnosed with PANDAS, Then catatonia Five+ mood episodes	Age 14 Slow onset Decreased toileting Decreased communication Decreased orientation Decreased feeding self No longer writes name	Mood episode in past year Two years since regression Moderate recovery of communication and self-care skills Less improvement with academics
Case 22 Female 15 years 0 month	Terminal deletion [62 kb] arr[hg18] 22q13.33(49,406,251-49,468,408)x1	Few single words Not toilet trained Needed help for dressing Fed self with utensils	None noted	Age 8 First episode manic, then depressive episodes 10+ episodes	No lasting loss of skills	Mood episode in past year No regression
Case 23 Female 39 years 5 months	Terminal deletion [2.5 Mb] arr[hg18] 22q13.32q13.33(47,003,473-49,515,911)x1	Phrase speech with poor articulation Toilet trained Intact ADLs Read simple books Memory games Jigsaw puzzles Dot-to-dot coloring	None noted	Age 32 First episode depressed Then recurrent depressed episodes with severe anxiety, SIB Then mania with catatonia 10+ mood episodes	No lasting loss of skills	Mood episode in past year No regression
Case 24 Female 36 years 7 months	Terminal deletion [69 kb] arr[hg19] 22q13.33(51,128,324-51,197,766)x1	Phrase speech Toilet trained Dressed self Fed self Read simple text Wrote at a second grade level	Puberty	Age 15 First episode agitation, OCD Then catatonia and depression Then manic episodes 10+ mood episodes	Age 18 Decreased urinary continence Decreased independence for ADLs Lost all academic skills	No episodes in past year 18 years since regression No recovery
Case 25 Female 24 years 1 month	Terminal deletion – unbalanced translocation [6.0 Mb] 46,XX,der(22)t(22;acro)(q13.33;p12); arr[hg19] 22q13.31q13.33(45,156,254-51,197,725)x1	Single words Toilet trained Fed self Did chores	Multiple family health stressors High strep titers	Age 21 First episode depressed with agitation, photophobia, then catatonia, diagnosed PANDAS One episode	No loss of skills	No episode in 3 years No regression
Case 26 Male 18 years 1 month	Sequence variant–Frameshift NM_033517.1:c.4649_4653dupGCAGC, p.Pro1552Alafs*14 dn (based on the results of his monozygotic twin brother)	Spoke in full sentences Toilet trained Dressed with assistance Fed self Read fluently Acted out plays Used computer with sophistication (e.g., booked travel)	Strep infection	Age 13 First episode manic with acute onset insomnia, tics, anorexia diagnosed PANDAS Then new pica, catatonia Then mutism and aggression associated with recurrence of manic symptoms 10+ mood episodes	Age 13 Lost language Decreased fine motor skills Decreased toileting Decreased utensil use Decreased interest in people and hobbies Destructive Sleep disrupted	Mood episode in past year Five years since regression Minimal recovery Slightly improved language and toileting Remains socially withdrawn Has not regained fine motor skills
Case 27 Male (twin of case 26) 18 years 1 month	Sequence variant – Frameshift NM_033517.1:c.4649_4653dupGCAGC, p.Pro1552Alafs*14 dn	Phrase speech Toilet trained Needed help with dressing Fed self with fingers Used computer Few sight words	Strep infection	Age 13 First episode manic with acute onset insomnia, tics, anorexia diagnosed PANDAS Then depression and catatonia 10+ manic and depressed episodes	Age 13 Lost most language Incontinent Decreased feeding self Decreased interest in people, activities	Mood episode in past year Five years since regression Moderate recovery Has regained some fine motor skills and social interests, but not language and full continence
Case 28 Female 26 years 3 months	Terminal deletion [70 kb] arr[hg19] 22q13.33(51,127,898-51,197,838)×1	Spoke in full sentences Toilet trained Independent with dressing and care Took own meds Black belt in karate	Menses	Age 16 Gradual onset aggression Then manic episodes with unsafe behaviors 10+ manic episodes	Age 18 Became confused about daily tasks and had trouble with self-care Language deteriorated Episodes of running away	Manic episode in past year Six years since regression Has recovered all skills
Case 29 Female 16 years 7 months	Sequence variant – Frameshift NM_033517.1:c.3679dupG, p.Ala1227Glyfs*69 dn	Spoke in full sentences Toilet trained Dressed self Independent for hygiene Fed self Read at 2nd grade level	Menses Hashimoto’s encephalopathy	Age 12 Gradual onset confusion and choking spells unrelated to food Then episodes of mania and OCD with catatonia and disorganized behavior and new behavior posing risk of accidental injury 10+ mood episodes	Age 12 Lost all adaptive skills, including continence and speech Lost all academic skills	Manic episode in past year Four years since regression Minimal recovery of relatedness and communication No recovery of academic skills Dependent for ADLs Requires constant supervision Appears to understand language but little speech Plays simple computer games
Case 30 Female 16 years 8 months	Terminal deletion with interstitial duplication [6.4 Mb] 46,XX,del [22](q13.3); arr[hg18] 22q13.31(43,276,438-49,691,432)x1, 22q13.31(42,835,935-43,272,271)x3	Minimal language Toilet trained for urine in daytime Fed self with fork Could put on clothes if in correct position	Mycoplasma pneumonia	Age 14 First episode depressed with catatonia Then OCD and manic symptoms Five+ mood episodes	Age 15 Lost daytime continence Lost interest in play and relatedness Decreased feeding self Decreased vocalization	Mood episode in past year Three years since regression Moderate recovery Has regained energy and vocalization, not daytime continence
Case 31 Female 49 years 9 months	Terminal deletion [5.0 Mb] (5 Mb terminal deletion with the breakpoint at 22q13.31)	Spoke a few words Toilet trained Fed self Ambulatory	None noted	Age 14 First episode manic Then repeated mood episodes at intervals of five or more years Three+ mood episodes	Age 47 Loss of skills with each psychiatric episode; recovered each time until age 47, then lost all language Lost ability to walk Lost feeding skills	Two years since last mood episode; Two years since regression No recovery No language Incontinent Cannot stand or walk, has difficulty maintaining sitting Cannot feed self Needs total care
Case 32 Female 31 years 11 months	Terminal deletion [76 kb] 46,XX; arr[hg19] 22q13.33(51,121,452-51,197,838)x1 dn	Spoke in full sentences Toilet trained Intact ADLs Planned recipes and cooked Worked with young children Ran with running group Volunteered at nursing home	None noted	Age 14 First episode anxiety/OCD Then mania Then depression with slow recovery over 2 years followed by severe mood cycling years later Three+ mood episodes	No loss of skills	Mood episode in past year (resolving) No regression
Case 33 Female 41 years 0 month	Terminal deletion [481 kb] arr[hg18] 22q13.33(49,210,245-49,691,432)x1	Single words Never toilet trained Fed self Undressed and pulled on pants Read sight words Poured drinks Rode tricycle Played with dolls	None noted	Age 7 First episode bizarre behavior with agitation, disorientation and insomnia (possible brief psychotic episode) Then OCD and new pica, followed by mania 10+ mood episodes	Age 16 Lost all meaningful speech; repeats words Unable to feed self, eats by lowering face to plate Unable to assist with dressing Lost all play Elopes frequently	Mood episode in past year 25 years since regression No recovery
Case 34 Female 17 years 8 month	Terminal deletion [53 kb] arr[hg19] 22q13.33(51,144,903-51,197,838)x1	Spoke in full sentences Toilet trained Fed self Dressed self Retrieved and poured cereal Did puzzles Played with Legos	Multiple social stressors	Age 14 First episode depressed with anorexia and incontinence Then mania and new pica Five+ mood episodes	Age 14 Decreased speech Decreased play Decreased dressing Decreased continence	No episode in past year Four years since regression Moderate recovery of language, self-care and play skills
Case 35 Female 23 years 1 month	Sequence variant – Frameshift NM_033517.1:c.4086_4087delAC, p.Arg1363Glnfs*31	Phrase speech with echolalia Toilet trained Fed self Poured milk Dressed self with assistance Needed help with ADLs Pretend play	Multiple social stressors	Age 12 First episode depressed with confusion, new incontinence and catatonia Then mania Three+ mood episodes	Age 12 Steady ongoing regression Lost all speech Incontinent Lost all self-care skills	Mood episode in past year 11 years since regression No recovery Requires constant supervision Only able to walk short distances with difficulty Cannot climb stairs
Case 36 Female 15 years 0 month	Sequence variant – Frameshift NM_033517.1:c.4065_4066delTG, p.Val1357Glyfs*4 dn	Spoke in full sentences Toilet trained Dressed self Fed self Read sight words Used tablet Math at 1st grade level	Social stressors	Age 14 First episode catatonic with urinary retention, insomnia and pica; did not recognize family; paranoid One episode	Still recovering from first episode with catatonia; cannot yet determine new baseline or whether lost skills meet criteria for regression	Eight months since onset of psychiatric illness Continues to regain language, personality, and orientation Still not able to open and use hands for ADLs Intermittently incontinent
Case 37 Female 17 years 5 months	Sequence variant – Nonsense NM_033517.1:c.4333C > T, p.Gln1445* dn	Phrase speech Toilet trained Fed self Dressed self with assistance Imaginative play Wrote name Copied text Drew pictures	Menses End of school year	Age 15 First episode depressed with insomnia, severe separation anxiety, apathy, decreased speech and interaction, swallowing difficulties, and catatonia One mood episode	Age 15 Lost most speech Lost ability to chew food Increased dependence for ADLs Lost all academic skills Lost imaginary play	No episode in past year Two years since regression Moderate recovery Regained some speech Eats normally Mostly continent Continues to not have academic skills, drawing skills, or imaginary play skills
Case 38 Female 16 years 9 months	Sequence variant – Frameshift NM_033517.1:c.2763delG, p.Pro922Argfs*34 dn	Spoke in full sentences Toilet trained Sociable	Menses UTI	Age 13 First episode hypomanic Then 2 years later manic with aggression, incontinence; anxiety, talking to self, compulsions, then catatonia Three manic episodes	Age 15 Lost all speech Lost ability to feed self Incontinent Decreased social interaction	No episode in past year One year since regression Substantial recovery Recovered language Recovered feeding Improved social interaction Continues with intermittent incontinence

*Abbreviations*: *ADLs* activities of daily living, *dn* de novo, *dx* diagnosis, *ECT* electroconvulsive therapy, *ICU* intensive care unit, *OCD* obsessive-compulsive disorder, *PANDAS* pediatric autoimmune neuropsychiatric disorder associated with streptococcus, *PECS* Picture Exchange Communication System, *UTI* urinary tract infection, *URI* upper respiratory tract infection

^**1**^ All were ambulatory and able to feed themselves with utensils prior to psychiatric illness

^**2**^ Cases 11, 14, 17, 22, 30, 33, and 35 had regression in speech, play, and social interaction before age 8, as well as subsequent regressions accompanying psychiatric illness at an older age

^3^ Regression defined as loss of skills that persisted at least 6 months after resolution of acute psychiatric episode

^**4**^ Regression occurred years after onset of psychiatric illness

^5^ “Symptoms” used when sub-threshold for diagnosis of either depressive or manic episode

As compared with the 509 PMSIR registrants not in this study who had genetic data available, sequence variants in *SHANK3* were present at higher rates in our sample (39% [15/38] vs 6% [32/509]; Fisher’s exact test, *p* = 4 × 10^− 8^), and conversely, terminal deletions were less frequent (61% [23/38] vs 92% [467/509]; Fisher’s exact test, *p* = 8 × 10^− 7^). Comparisons with 130 PMSIR registrants of similar age with questionnaire data available are also reported in Table 1. In addition, deletions tended to be smaller in the participants of this study than in the PMSIR (mean size 1.634 Mb vs 3.633 Mb; unpaired two-tailed Student’s *t* test, *p* = 0.0019). In particular, individuals with small deletions, defined as those including at most the four distal genes, *ARSA*, *SHANK3*, *ACR*, and *RABL2B*, are over-represented among study participants compared to the PMSIR (57% [12/21 deletions with size information] vs 19% [72 /387]; Fisher’s exact test, *p* = 1.6 × 10^− 4^). (Heterozygous loss of *ARSA*, *ACR*, and *RABL2B* does not contribute to the PMS phenotype, so deletions including these genes are equivalent to deletions involving only *SHANK3.*)

### Developmental history

Although documentation of the severity of ID was not available, reported levels of adaptive functioning suggest that mild to moderate ID was common in this sample prior to the onset of psychiatric symptoms and regression and that these individuals were less impaired prior to their psychiatric illness as compared to the PMSIR reference sample (Table 1). Prior to the onset of psychiatric illness, study participants were significantly more likely than participants in the PMSIR sample to ever have walked independently, achieved toilet training, verbal expression with at least phrase speech, and independence with dressing.

### Psychiatric illness

Psychiatric difficulties began between ages 10 and 18 in the majority of cases (28/38; 74%). Psychiatric symptoms emerged between the ages of 7 and 10 years in 5/38 cases (13%), while 5/38 cases (13%) developed psychiatric symptoms between ages 21 and 32. Using the DM-ID-2, first episodes met criteria for a manic episode in 17/38 cases (45%) and for a depressive episode in 14/38 cases (37%). First episodes in the remaining cases included six (16%) with a mixture of mood and anxiety symptoms, and one case of disorganized, bizarre behavior that suggested a brief psychotic episode.

Most participants (27/38; 71%) had experienced a mood episode in the year before the study. The aftermath of acute episodes was associated with higher baseline levels of anxiety and irritability and with regression. Subsequent psychiatric episodes were superimposed and caregivers clearly distinguished episodes from baseline states. First episodes ranged from 8 months to 35 years before study participation. Reported episode length varied from periods of days to months. Most cases (34/38; 89%) had multiple distinct episodes consistent with a mood disorder, including 12 of 38 (32%) with two to four distinct episodes, 6 of 38 (16%) with five to nine episodes, and 16 of 38 (42%) with more than ten episodes (see Table 2 for details). Few cases (4/38; 11%) reported only a single psychiatric episode; all four of these were within the 3 years prior to study participation.

Twenty-nine of 38 cases (76%) reported a manic episode at some point in their illness; 24 of 38 (63%) reported both manic and depressive episodes. Nineteen of 38 cases (50%) were reported to have had psychotic symptoms during an acute mood episode. One participant presented with both depressive and manic episodes and with psychosis in between mood episodes, suggesting schizoaffective disorder.

New anxiety symptoms accompanied mood episodes in the majority of participants (26/38; 68%). Six participants (16%) met criteria for obsessive-compulsive disorder at some point in the course of their illness. Four participants (11%) met criteria for generalized anxiety disorder. Only two participants (5%) had first episodes that were predominantly anxious.

Symptoms consistent with a diagnosis of catatonia associated with another mental disorder were reported in 20 of 38 cases (53%). Seven of the 20 (35%) were on neuroleptics at the time of emergence of catatonic symptoms. Cases with sequence variants in *SHANK3* met diagnostic criteria for catatonia significantly more often (12/15; 80%) than cases with terminal deletions (8/23; 35%) (Fisher’s exact test, *p* = 0.009).

### Regression

The interview explored both abilities lost and the degree of return of skills over time. Caregivers were asked which skills were lost, at what age, and which, if any, skills were regained. Seven of the participants (18%) had experienced regressions in early childhood affecting communication, social interaction, and/or imaginative play (five by age two, one at age four, one at age seven), but we do not elaborate on those early regressions in the current report. Analysis focused on the 25 of 38 cases (66%) who had regressions that began within the 3 years after the onset of psychiatric episodes. Most of these regressions (21/25; 84%) began within a year after the onset of psychiatric episodes and involved multiple domains of function. Descriptions of the areas of skill lost and recovery are summarized in Table 2, which includes developmental status in terms of speech, toilet training, ability to dress and wash self, academic skills, and vocational activities when present. Caregiver reports of recovery of skills ranged from continuing loss of further skills to complete return to baseline function before the onset of psychiatric symptoms. Overall, more than half of the participants who regressed in the 3 years after the onset of psychiatric illness reported minimal recovery (14/25; 56%).

Antecedents to the onset of psychiatric illness and regression were explored in the interview. Menstrual cycling was reported to play a triggering role in 11 of 31 (35%) of the females. Acute infections were suspected triggers in 11 of 38 cases (29%). Psychosocial stressors were suspected triggers in 10 of 38 cases (26%). Several participants reported differing antecedent stressors for different episodes.

### Co-morbid conditions

Only a quarter (9/38; 24%) of study participants was known to have PMS before the onset of psychiatric symptoms and associated regression. Sixteen cases (42%) had diagnoses of ASD before the onset of psychiatric symptoms and regression; five more received an ASD diagnosis after the onset of psychiatric illness and regression.

Fifteen of 38 cases (40%) reported one or more afebrile seizure. Eight of those 15 cases (53%) reported a seizure in the 2 years preceding the interview. Fifteen of 38 cases (40%) had weight loss of 10 to 25 kg accompanying psychiatric episodes. Chronic constipation, intermittent urinary incontinence, and episodes of acute urinary retention were prevalent in this sample, particularly as subjects aged. Chronic constipation was noted in 32 cases (84%), with episodes of acute urinary retention reported in 18 (47%). The discomfort associated with these conditions may lead to non-specific behaviors such as agitation, aggression, and self-injury in people with ID; these chronic symptoms were not coded as mood episodes. A small subset of subjects (4/38; 11%) had an immune-related disorder. Two participants (5%) were diagnosed with Hashimoto’s thyroiditis, one of whom was diagnosed with Hashimoto’s encephalopathy, and two participants (5%) had immunoglobulin deficiency, including one with common variable immunodeficiency.

### Therapeutic interventions

At the time of interview, 32 of 38 participants (84%) were receiving one or more psychiatric medications, with 14 of 38 (37%) receiving four or more psychiatric medications. More than half (21/38; 55%) took anticonvulsants; all of these had mood symptoms, while 12 also had a history of seizures. Over half (55%) were receiving neuroleptics. Benzodiazepines were used either regularly or on an as-needed basis in 16 of 38 cases (42%). Alpha agonists, beta-blockers, antihistamines, and trazodone were prescribed for sleep or aggression. Smaller numbers of participants were receiving selective serotonin reuptake inhibitors (SSRIs), tricyclic antidepressants, buspirone, lithium, cannabinoid oil, or n-acetylcysteine.

Full details of dosages and adjustments to treatment regimens in response to changing psychiatric status were inconsistently available and are therefore not reported. However, many parents kept extensive notes, and their reports of responses to medication trials suggest two patterns worth noting: (1) participants were reported to be sensitive to side effects from neuroleptics, with adverse events including extrapyramidal symptoms, worsening aggression and/or onset of catatonic symptoms reported in 14 of 30 cases (47%), while (2) seven of nine participants (78%) treated with SSRIs for depressive symptoms developed agitation, aggression, or other manic-like symptoms within weeks of the start of treatment.

Three individuals were treated with electroconvulsive therapy (ECT) for catatonia with reported significant benefit; two continue to require maintenance ECT (duration of treatment 18 months in one, > 8 years in the other) while the third discontinued after initial response due to agitation. One participant with recurrent catatonia on maintenance ECT was reported to have also benefited from intravenous immunoglobulin (IVIG) with improved cognition and motivation, but developed aseptic meningitis and a rash after the fifth treatment, leading to discontinuation of IVIG.

Nine participants (26%) were reported to have received immunomodulatory treatments for diagnoses of pediatric autoimmune neuropsychiatric disorders associated with streptococcal infections (PANDAS) syndrome or of autoimmune encephalopathy. Six were diagnosed with PANDAS syndrome on the basis of clinical symptoms and positive strep cultures or elevated titers. One participant was diagnosed with Hashimoto’s encephalopathy, and two with seronegative autoimmune encephalitis. Treatments included (1) antibiotics, (2) IVIG, and/or (3) systemic anti-inflammatories such as nonsteroidal anti-inflammatory drugs, steroids, or rituximab.

In four of six cases diagnosed with PANDAS syndrome, parents reported that antibiotic treatment was effective in reducing or eliminating acute symptoms. The remaining two improved slowly; one also received IVIG and the other received lorazepam; these parents were not confident that antibiotics had played a role in recovery. Treatment for PANDAS symptoms did not appear to prevent future mood episodes or regression, as all but one of these cases went on to have further psychiatric episodes and some had later regressions. The one participant diagnosed with Hashimoto’s encephalopathy continues to receive regular infusions of IVIG with steroids, as well as rituximab. The two participants with diagnoses of seronegative autoimmune encephalopathy receive monthly IVIG.

Eight females were on hormonal therapy to eliminate menstrual cycles and reduce associated mood symptoms. Non-pharmacological treatments varied widely by geographic location and age, with those still in school more likely to be receiving structured behavioral interventions. Data on past and current behavioral programs and therapies were not included in the interview.

## Discussion

This case series has characterized a subset of individuals with PMS who have episodic, severe neuropsychiatric illness, frequently beginning in adolescence or early adulthood, with major impacts on functional status. The symptoms and course of illness often closely resembled bipolar illness, with psychotic features almost exclusively present in the context of mood episodes. None of the participants in our sample presented with a primary psychotic disorder, such as schizophrenia. However, one participant presented with distinct psychotic and affective episodes characteristic of schizoaffective disorder. Using the DM-ID-2 criteria [30], all but one of the participants had either a mood disorder or an anxiety disorder, with multiple discrete periods of illness in all but four.

Several triggers were often reported as temporal antecedents to the onset of psychiatric changes. Biological triggers included infections and changes in hormonal status, while environmental factors included stressful life events. Similar patterns have been observed in other, more common neurogenetic syndromes, including Down syndrome [32], Williams syndrome [33], and 22q11.2 deletion syndrome [34].

It is critical to emphasize the high incidence of catatonic symptoms in this subset of individuals with PMS, as catatonia often goes unrecognized or undertreated in individuals with developmental disabilities [25, 35]. Recognition and treatment of both stuporous and hypermotoric catatonia is crucial, as symptoms can lead to life-threatening complications. Other genetic conditions involving copy number variants, such as 22q11.2 deletions [36], have also been associated with psychiatric symptoms and with catatonia. Similar to their use in treating catatonia in other conditions, benzodiazepines and ECT were used in this cohort with reported tolerability and effectiveness.

Regression has long been recognized in neurodevelopmental disorders such as ASD [37], but the triggers and mechanisms are not well understood, and the literature focuses largely on early childhood regression, especially as it relates to Rett syndrome [37–40]. Significant cognitive and behavioral regression has been documented in PMS [5, 6, 9, 15, 16, 18, 41]. Verhoeven et al. [20] have recently published on the course of illness of 24 adolescents and adults with PMS referred for evaluation and treatment of “challenging behaviors and unstable mood.” In that sample, there were five individuals with periodic catatonic symptoms, and four other individuals who developed progressive loss of skills in their third or fourth decade.

Two thirds (66%) of our sample described lasting regressions that began within 3 years of the onset of psychiatric illness. These regressions in communication, self-care, and motor functions left a subset of previously more capable individuals largely non-verbal, incontinent, and unable to dress or feed themselves. Many of these participants continue to experience episodic psychiatric illness in addition to developmental decline. Interestingly, a similar combination of rapid onset psychiatric disturbance with marked regression and catatonia has been described in Down syndrome [42, 43] and subsequently labeled “Down syndrome disintegrative disorder.” A recent case series [44] documented a role for immunotherapy in restoring function and stability in these individuals, although it is also recognized that cognitive decline in Down syndrome relates to amyloid precursor protein gene triplication [45–47]. Regression in PMS may be more common than in Down syndrome, and its potentially devastating impact warrants ongoing study of the natural history, mechanism, and targets for intervention.

Given the methodological limitations and sample selection bias in this study, we are unable to draw conclusions about the prevalence of psychiatric illness and/or regression among people with PMS. This study was also limited by the lack of standardized or validated tools to measure neuropsychiatric symptoms in severely developmentally disabled children and adults. However, within this self-referred subset of individuals with PMS whose caregivers identified psychiatric illness, we observed a distinct pattern of affective episodes, with onset typically in puberty, a strikingly high incidence of catatonic symptoms, and an association with substantial regression in the second through fourth decades of life. Of note, a recent literature review analyzed prior reports of 56 individuals with PMS with accompanying neuropsychiatric symptoms, and concluded that “clinicians and caregivers need to be vigilant for loss of skills and neuropsychiatric changes in adolescents and adults with PMS, including the development of bipolar disorder and catatonia” [48].

Individuals with PMS who present with new psychiatric symptoms require careful monitoring and cautious early intervention. Response to traditional psychiatric interventions is variable, and the role for immunomodulatory treatments and ECT in specific subsets of patients should be explored in future studies. The pattern of rapid and severe deterioration described in more than half of this group calls for further investigation to identify triggers and the underlying biology and to delineate possible strategies for prevention and timely intervention. While the current study does not allow for specific treatment recommendations, there is an urgent need for clinical trials to assess the efficacy of existing psychopharmacological treatments for psychiatric symptoms in PMS, in addition to developing novel, targeted therapeutics.

The preponderance of females in this sample (> 4:1) is notable and calls for replication. Sequence variants in *SHANK3* were also six times more common in this sample than in the PMSIR. These findings raise questions about whether psychiatric problems and regression disproportionately affect females and/or individuals with *SHANK3* sequence variants, in contrast to those with deletions. In addition, prior to the onset of psychiatric illness, most of the participants described herein were less severely impaired in their adaptive functioning than is reported in most studies of PMS. This finding is likely related to the high proportion of individuals with *SHANK3* variants and small deletions in this sample.

Further studies should attempt to clarify if psychiatric difficulties are actually more common in individuals who are more verbal and social and who have more intact adaptive functioning at baseline, or, whether this finding is the result of selection bias in our sample, as the majority of participants (76%) were only diagnosed with PMS after the onset of psychiatric symptoms.

Finally, our sample underscores the importance of genetic testing as part of the medical work-up for individuals with this presentation. Chromosomal microarray is recommended for the evaluation of all children with global developmental delay or intellectual disability [49]. In the setting of superimposed psychiatric illness or regression, chromosomal microarray should be considered as first tier testing, followed by sequencing of *SHANK3* if the microarray is unrevealing.

## Conclusion

This study confirms that individuals with PMS are at risk of developing severe neuropsychiatric illness in adolescence or early adulthood. In most cases, the symptoms appear consistent with the expression of bipolar disorder in individuals with intellectual disability, with catatonia noted as a common co-occurring condition. Triggers may include infections, changes in hormonal status, and stressful life events. Significant cognitive and behavioral regression beyond a baseline level of disability has been previously reported in PMS and accompanied the onset of psychiatric illness in a majority of the cases reported here. Our results must be interpreted with caution given the potential selection bias in recruitment, but it is clear that people with PMS may show a rapid and severe deterioration that requires careful monitoring and intervention. Our findings also highlight the relevance of genetic testing in the work-up of individuals with intellectual disability and acute psychiatric illness or regression. Future research is needed to clarify the prevalence and nature of psychiatric symptoms and regression among larger unbiased samples of individuals with PMS and to delineate any shared mechanisms with other neurodevelopmental disorders presenting with psychiatric illness and/or regression in adolescence or early adulthood. Identification of early clinical and biological markers would contribute to our understanding of the underlying neurobiology of these disorders and potentially aid in monitoring, early intervention, or prevention.

## Additional files

Additional file 1. Caregiver Interview for Psychiatric Illness in Persons with ID (CIPIPID)

Additional file 2. Adapted Diagnostic Criteria, DM-ID-2

## Abbreviations

ASD: Autism spectrum disorder
DM-ID-2: Diagnostic Manual - Intellectual Disability, Second Edition
ECT: Electroconvulsive therapy
ID: Intellectual disability
IVIG: Intravenous immunoglobulin
PANDAS: Pediatric autoimmune neuropsychiatric disorders associated with streptococcal infections
PMS: Phelan-McDermid syndrome
PMSIR: Phelan-McDermid Syndrome International Registry
