# Supplementary material for: Psychiatric illness and regression in individuals with Phelan-McDermid syndrome
Source: J Neurodev Disord. 2020 Feb 12;12:7. doi: 10.1186/s11689-020-9309-6 (PMC7014655; doi:10.1186/s11689-020-9309-6)
Supplement: Supplementary file 1 — Caregiver Interview for Psychiatric Illness in Persons with ID (CIPIPID) [file 11689_2020_9309_MOESM1_ESM.docx]

**Caregiver Interview for Psychiatric Illness in Persons with ID (CIPIPID)**

_______________________________ _________________________

Patient Name Patient Date of Birth

_______________________________ _________________________

Name of interviewee Relationship to Patient

_______________________________ _________________________

Name of Interviewer Date of Interview

___________________________________________________________________

Indicate the best email and phone number to contact the interviewee for follow-up

**Introduction:**

Start by inviting the caregiver to “tell the story” of the evolution of the person’s development, and the onset and course of psychiatric illness. Confirm that they understand that you will be taking notes, and that they will have an opportunity to review your notes, and correct them. Most caregivers will have a narrative that they are eager to share, which will provide a wealth of context and detail. You will then be able to circle back and fill in details, guided by the questions below.

Set the stage with an invitation such as: “the goal of this interview is to learn about this person’s development and the psychiatric issues that they have faced. We will talk about a lot of different areas, and it would be helpful to start with what you see as the big points along the road”.

Listening carefully to their story -- and asking questions to clarify and confirm that you have understood correctly -- helps to establish trust that will facilitate the depth of the interview, and provides support to the caregiver in sharing what are often quite painful histories. If the caregiver does not respond to the invitation to tell you their story, you can begin by asking the questions. Follow the caregiver’s lead; you may find that you return multiple times to the list of questions, filling in different areas as the interview proceeds.

NOTE: If the caregiver is not a family member of the patient, some of these questions need to be adapted or left out altogether.

How did the pregnancy and delivery go?

(If the caregiver is not the parent, ask what they know about any pregnancy or newborn issues).

Were there any concerns at birth or in the first weeks of life?

(if yes, what were they, how did they evolve)

What was this baby like in terms of his or her rhythms and temperament?

Feeding?

Sleeping?

Did you have to develop any elaborate maneuvers to soothe the baby or get the

baby to sleep?

Did the baby look at faces and interact as expected?

Did the baby enjoy being held?

Did the baby grow well?

At what age did the child

crawl

walk

babble

start to communicate in words or signs

toilet train (distinguish age trained for urine vs bowel movements)

When did someone first have concerns about the child’s development?

Who had those concerns?

What were those concerns?

What sort of evaluations did the child get? How old was the child then?

What did you understand the evaluations to show? (only for parent interviews)

What did the doctors think the issue was at that point? (only for parent interviews)

When did the child start in school, and how did that go?

Talk through the school years… did the child start out in mainstream education?

Was there a change to specialized classrooms?

What services did the child receive, and how much progress did they make in reading, writing, speaking, mathematics, and self-care and life skills?

Do you know the results of any IQ tests? Do those results make sense to you?

Does the patient have an autism diagnosis, and if so, at what age was it given, and by whom?

Does the patient have any psychiatric diagnoses? If so, at what age were they given, and by whom?

If the patient has a specific diagnosis that accounts for their developmental disorder, what is it, and how did you learn of it?

How old was the patient when he or she was most able to do things for their self and to understand and communicate?

(Transcribe a brief narrative description of their function at this best stage)

At what age did emotional/behavioral problems begin?

What sort of emotional/behavioral problems?  How have they evolved over time?

Are they constant issues, or do they come and go for weeks or months at a time?

Clarify any baseline behavioral challenges that can be viewed separately from acute psychiatric symptoms. These might include difficulties with sleep, stimming behaviors, impulsive aggression or bolting, eating non-food items, difficulties with self-care, etc.

In the following section, you are exploring for symptoms of psychiatric disorders.

For any area in which the caregiver reports distinct symptoms, note how many distinct periods of disturbance have occurred in the patient’s life, how long each episode lasted, and any observations caregivers have about the way those episodes ended (gradual, quickly, response to interventions).

In some cases episodes will be too frequent to capture each one separately, for example some women may have mood disturbance with each monthly cycle; in such cases, a summary that captures the pattern is sufficient.

Has he or she had:

* Periods of distinctly different mood, energy, or behavior such as:

LOW SPELLS -- stretches of days or weeks in which they may have some of the following symptoms:

- sleep much more and are inactive, tired, or listless compared to their normal self;
- are tearful, have a lack of interest in things that usually gave them pleasure, or new social withdrawal
- show a marked change in appetite or weight
- are irritable, may have new sensitivity to sounds or lights

If yes, review diagnostic criteria for depression

HIGH SPELLS – stretches of days or weeks in which they they may have some of the following symptoms:

- Have a lot less need for sleep or ability to stay asleep,
- Have a marked change in appetite and weight,
- Are agitated and moving all the time, or have increased energy and physical activity
- Talk or vocalize more than usual, or make loud, silly or giddy vocalizations
- Have an intense new focus on getting things done (examples: taking everything out of closets, making objects, giving things to people, changing clothes repeatedly)
- Engage in unexpected, abrupt, difficult to manage behaviors

If yes, review diagnostic criteria for mania

WITHDRAWN/ DISENGAGED SPELLS - Periods of decreased response to people, decreased self-feeding, not moving around, changes in the way they hold or move their body, new repeating of words, new and unusual bathroom behavior, drooling, odd facial expressions, copying motions or speech of other people (if yes, review diagnostic features of catatonia)

DISORIENTED/PSYCHOTIC SPELLS - Hallucinations, delusions or other, new disorganized or confused behavior

ANXIETY SPELLS - Periods of NEW intense anxiety, repeated questioning or motions, panic, obsessions, or compulsive behavior

SELF-INJURY - Behavior that hurts their own body on purpose (hitting their own body, pushing on their eyes, picking at skin until it bleeds, etc)

INJURY TO OTHERS - Hurting other people on purpose or aggression

SOMETHING ELSE - Is there any other pattern of concern to you that I didn’t ask about?

If yes to any of the questions above, explore in detail

- the onset (abrupt, insidious)
- evolution
- duration
- resolution
- recurrence

Did these problems start around the time of puberty?

If female, were symptoms related to menstrual cycles?

Were there medical illnesses that you felt might have triggered problems?

Were there any stresses in the person’s or family’s life, or significant changes in relationships, that you felt were triggers for the problems? (This might include changes in school or day programs, or a sibling growing up and leaving home, or a favorite adult leaving).

If he or she has been treated for these problems, what treatments have been tried, and how did the person respond? (list as many as the caregiver can remember)

What treatments are now being used?

Did this person ever have seizures?

If yes, what sort of seizures, how have they been managed, what tests (EEG, imaging, lumbar puncture, etc.) have been done, and what were the findings?

Were there any other medical problems?

What specialists currently see the patient, and what medications do they now take?

Has he or she lost skills in a significant way?

If yes, when, which skills, at what age, and to what extent were those skills ever regained?

Was the loss gradual/steady, or in chunks with plateaus in between?

Ask about how the person communicates, their ability to navigate their environment, toileting, ability to dress and wash self, self-feeding with or without utensils, any home chores, any academic skills such as reading sight words or text, doing simple math, using an iPad or computer, performing any community jobs, participating in any teams, hobbies or community activities.

Transcribe a brief narrative description of current function

Where does the child live? With family? In adult foster care? In group care?

Is there anything else that you would like me to know, that I have not asked you about?
