# Supplementary material for: Psychiatric illness and regression in individuals with Phelan-McDermid syndrome
Source: J Neurodev Disord. 2020 Feb 12;12:7. doi: 10.1186/s11689-020-9309-6 (PMC7014655; doi:10.1186/s11689-020-9309-6)
Supplement: Supplementary file 2 — Adapted Diagnostic Criteria, DM-ID-2 [file 11689_2020_9309_MOESM2_ESM.docx]

**Additional File 2. Adapted Diagnostic Criteria, DM-ID-2**

| **Manic Episode** | |
| --- | --- |
| **DSM-5 Diagnostic Criteria** | **Applying Criteria for Mild to Profound Intellectual Disability** |
| A. A distinct period of abnormality and persistently elevated, expansive, or irritable mood and abnormally and persistently increased goal directed activity or energy, lasting at least 1 week and present most of the day, nearly every day (or any duration if hospitalization is necessary) | A. No adaptation |
| B. During the period of mood disturbance and increased energy or activity, three (or more) of the following symptoms (four if the mood is only irritable) are present to a significant degree and represent a noticeable change from usual behavior | B. For individuals who have limited expressive language skills, during the period of mood disturbance, adjust criteria to two (or more) of the symptoms listed below if present to a significant degree – three if the mood is only irritable |
| 1. Inflated self-esteem or grandiosity | 1. No adaptation.   **Note 1**: Observers may report that the individual with intellectual disability expresses: exaggerated claims of skills or accomplishments (based on developmental profile at baseline i.e., individual claims he has a car but does not, claims skills he doesn’t have such as ability to drive, states he is the director of the hospital), exaggerates social events (“I’m getting married” when not seeing anyone or not engaged), claims a relationship with a famous person, claims a relationship with a brief acquaintance, believes he is a super hero (not fantasies consistent with developmental profile)  **Note 2**: At preoperational cognitive stage of development, fantasy and reality are not distinguished. Claims may represent wishes versus mood congruent delusional beliefs |
| 1. Decreased need for sleep (e.g., feels rested after only 3 hours of sleep) | 1. No adaptation.   **Note**: Observers may report that the individual with intellectual disability sleeps 0 – 3 hours per night, goes to sleep much later than usual, wakes much earlier than usual, gets ready for the day very early. More difficulties may occur at night than previously reported. The individual may be doing usual daytime activities in the middle of the night. When sleeping less, there may be minimal signs of fatigue the next day. The individual may appear tired but cannot sleep except briefly, keeps active – seems “driven”. The sleep problem resists treatment and is a departure from baseline (the individual is not disturbed by noise at night, is not sleeping during the day, and does not have a lifelong history of poor sleep). |
| 1. More talkative than usual or pressure to keep talking | 3. No adaptation.  **Note 1**: Observers may report the following changes in regards to an individual with intellectual disability: increase in vocalizing, screaming, noise making, or talking; nonstop or very rapid vocalizing, etc.; asks repeated questions, doesn’t wait for answers, decreased ability to listen, frequently interrupts, increase in perseverations, engages in frequent monologues, singing loudly – increases in noise making or vocalizing or screaming that is nonverbal; all symptoms are a departure from usual either because the symptoms are new or much more intense and frequent (baseline exaggeration).  Note 2: Anxiety may also cause people with intellectual disability to talk fast or to talk or vocalize more. |
| 1. Flight of ideas or subjective experience that thoughts are racing | 4. No adaptation.  **Note**: Observers may report that the individual with intellectual disability: jumps rapidly from topic to topic, or states thing like “My thought are moving fast.” This must be a change from what is usual, not an individual who typically changes frequently when well. The clinician must also establish a baseline developmental profile from records/history. |
| 1. Distractibility (i.e., attention too easily drawn to unimportant or irrelevant external stimuli) as reported or observed | 5. No adaptation.  **Note**: Observers may report that the individual with intellectual disability shows a reduced productivity at work or day program, has diminished self-care skills, appears easily distracted or cannot complete tasks he or she used to be able to finish, has shown the onset of or increase in agitated behaviors when asked to do activities that require concentration, has apparent memory problems that “come and go”, has unexplained skill loss, shows an uncharacteristic inability to learn new skills as expected, has had to stop working or attending programs due to poor performance. These problems focusing attention are new and represent a change (are not life-long). The problems in concentration or completing tasks seem mostly due to not being able to finish what is started or stay with a project and because attention is easily drawn to noise or activity going on around the person. |
| 1. Increase in goal-directed activity (either socially, at work or school, or sexually) or psychomotor agitation i.e., purposeless non-goal directed activity.) | 6. No adaptation.  **Note**: Observers may report that the individual with intellectual disability: engages in activities in a “sped up manner”, rarely sits down, is up and down from seat a lot, paces, walks rapidly, seems “driven”, races around the room, has become very intrusive, is much more physically active than before, and can’t even sit long enough to eat. |
| 1. Excessive involvement in pleasurable activities that have a high potential for painful consequences (e.g., engaging in unrestrained buying sprees, sexual indiscretions, or foolish business investments) | 7. No adaptation.  Note: Observers may report that the individual with intellectual disability engages in much more sexual behavior or talk, reports more sexual activity than usual, masturbates frequently and much more than before, exposes self in public and this is usual, and is touching others in a sexual manner. |
| C. The mood disturbance is sufficiently severe to cause marked impairment in social or occupational functioning or to necessitate hospitalization to prevent harm to self or others, or there are psychotic features | C. No adaptation. |
| D. The episode is not attributable to the physiological effects of a substance (e.g., drug of abuse, a medication, other treatment) or to another medical condition.  **Note**: A full manic episode that emerges during antidepressant treatment (e.g. medication, electroconvulsive therapy) but persists at a fully syndromal level beyond the physiological effects of that treatment is sufficient evidence for a manic episode and, therefore, a bipolar 1 diagnosis. | D. No adaptation.  **Note 1**: For people with intellectual disability, residential, vocational or other program placements may be lost due to the acute mood episode.  **Note 2**: For people with intellectual disability, almost any physical problem that causes pain or distress may also cause difficulty-focusing attention, sleeping, eating and psychomotor agitation. Rapid changes in medications that may provoke irritability, agitation, sleep problems and withdrawal emergent motor restlessness (akathisia) that can mimic a manic episode.  **Note 3**: For people with severe/profound intellectual disability, there is even less likelihood of an accurate report from the individual regarding his or her physical distress. Informants may attribute agitated behaviors to the previously diagnosed psychiatric syndrome and miss a new medical problem. There may be a tendency to diagnose bipolar syndromes in people with intellectual disability who have presented with manic like symptoms only when treated with medications such as antidepressant SSRIs. |
| **Note**: Criteria A-D constitute a manic episode. At least one lifetime manic episode is required for the diagnosis of bipolar 1 disorder. |  |

Reprinted with permission from the Diagnostic and Statistical Manual of Mental Disorders, Fifth Edition, (Copyright 2013). American Psychiatric Association.

| **Hypomanic Episode** | |
| --- | --- |
| **DSM-5 Diagnostic Criteria** | **Applying Criteria for Mild to Profound Intellectual Disability** |
| A. A distinct period of persistently elevated, expansive, or irritable mood and abnormally and persistently increased activity or energy, lasting at least 4 consecutive days and present most of the day, nearly every day. | A. No adaptation.  **Note**: Observers may report that the individual with intellectual disability has been loud, inappropriately laughing or singing, excessively giddy, silly; intrusive, getting into other’s space; smiling excessively and in ways that are not appropriate to the social context. Elated mood may be alternating with irritable mood. In people with severe/profound intellectual disability, irritable mood may be more common. This must be a change from typical behavior for the person. |
| B. During the period of mood disturbance and increased energy and activity three (or more) of the following symptoms (four if the mood is only irritable) have persisted, represent a noticeable change from usual behavior, and have been present to a significant degree. |  |
| 1. Inflated self-esteem or grandiosity | 1. No adaptation.   See notes for Manic Episode above. |
| 2. Decreased need for sleep (e.g., feels rested after only 3 hours of sleep) | 1. No adaptation.   See notes for Manic Episode above. |
| 1. More talkative than usual or pressure to keep talking | 3. No adaptation.  See notes for Manic Episode above. |
| 1. Flight of ideas or subjective experience that thoughts are racing | 4. No adaptation.  See notes for Manic Episode above. |
| 1. Distractibility (i.e., attention too easily drawn to unimportant or irrelevant external stimuli) as reported or observed | 5. No adaptation.  See notes for Manic Episode above. |
| 1. Increase in goal-directed activity (either socially, at work or school, or sexually) or psychomotor agitation | 6. No adaptation.  See notes for Manic Episode above. |
| 1. Excessive involvement in pleasurable activities that have a high potential for painful consequences (e.g., engaging in unrestrained buying sprees, sexual indiscretions, or foolish business investments) | 7. No adaptation.  See notes for Manic Episode above. |
| C. The episode is associated with an equivocal change in functioning that is uncharacteristic of the person when not symptomatic | C. No adaptation. |
| D. The disturbance in mood and change in function are observable by others | D. No adaptation. |
| E. The episode is not severe enough to cause marked impairment in social or occupational functioning, or to necessitate hospitalization. If there are psychotic features, the episode is, by definition, manic | E. No adaptation. |

Reprinted with permission from the Diagnostic and Statistical Manual of Mental Disorders, Fifth Edition, (Copyright 2013). American Psychiatric Association.

| **Major Depressive Disorder** | |
| --- | --- |
| **DSM-5 Diagnostic Criteria** | **Applying Criteria for Mild to Profound ID** |
| A. Five (or more) of the following symptoms have been present during the same 2-week period and represent a change from previous functioning; at least one of the symptoms is either (1) depressed mood or (2) loss of interest or pleasure  **Note**: Do not include symptoms that are clearly attributable to another medical condition | A. Four (or more) symptoms have been present during the same 2-week period and represent a change from previous functioning: At least one of the symptoms is either (1) depressed mood or (2) loss of interest or pleasure, or (3) irritable mood.  **Note**: Do not include symptoms that are clearly attributable to another medical condition |
| 1. Depressed mood most of the day, nearly every day, as indicated by either subjective report (e.g., feels sad or empty) or observation made by others (e.g., appears tearful). (Note: In children and adolescents, can be irritable mood). | 1. Depressed or irritable mood most of the day, nearly every day, as indicated by either subjective report or observation made by others.  **Note**: In people with ID, depressed mood may be described by others in one or more of the following ways, that constitutes a change from what is usually observed in this individual: sad facial expression, flat affect or absence of emotional expression, rarely smiles or laughs, cries or appears tearful **Note**: Observers may describe individuals with ID who are irritable as: appearing grouchy or having an angry facial expression, having the onset of (or increase in) agitated behaviors (assaults, self-injurious behavior, spitting, yelling, swearing, disruptive or destructive behaviors) accompanied by angry affect. |
| 1. Markedly diminished interest or pleasure in all, or almost all, activities most of the day, nearly every day (as indicated by either subjective account or observation). | 2. No adaptation.  **Note**: Observers may describe individuals with ID: refuses preferred activities, appears withdrawn, spends excessive time alone (more time than before), participates but shows no signs of enjoyment, becomes aggressive in response to request to participate in activities he or she used to like, has lost response to reinforces, finds previously motivating events or objects no longer motivating, avoids social activities, aggresses or becomes agitated when prompted to attend social activities once enjoyed. |
| 1. Significant weight loss when not dieting or weight gain (e.g., a change of more than 5 percent of body weight in a month), or decrease or increase in appetite nearly everyday (Note: In children, consider failure to make expected weight gains.) | 3. No adaptation.  **Note**: Observers may report the individual with ID: is eating in excess, is obsessing about food, is stealing food, is refusing meals, has experienced recent weight loss or gain, exhibits agitated behaviors emerge during meal times or in relation to food (throws food on floor, screams when meal arrives) |
| 1. Insomnia or hypersomnia nearly every day | 4. No adaptation.  **Note**: Observers may report the individual with ID: has difficulty falling asleep, awakens in the early morning, sleeps excessively, has shown a recent increase in problem behaviors late at night, very early in the morning, takes frequent naps, falls asleep during the day, is up and down all night, sleeps very little at night and seems tired |
| 1. Psychomotor agitation or retardation nearly every day (observable by others, not merely subjective feelings of restlessness or being slowed down) | 5. No adaptation.  **Note**: Observers may report the individual with ID: rarely sits down, is up and down from a seat a lot, paces, walks rapidly, fidgets, has slowed movements, has decreased or stopped talking, vocalizes much more or less than usual, is much less physically active than before |
| 1. Fatigue or loss of energy nearly every day | 6. No adaptation.  **Note**: Observers may report the individual with ID: appears tired or reports feeling tired, refuses or becomes agitated about activities that require physical effort, spends excessive amounts of time just sitting or excessive amounts of time lying down, has dark circles under eyes |
| 1. Feelings of worthlessness or excessive or inappropriate guilt (which may be delusional) nearly every day (not merely self-reproach or guilt about being sick) | 7. No adaptation.  **Note**: Observers may report the individual with ID: makes negative self-statements; identifies self as a “bad” person; often expects punishment, without a history of harsh treatment; blames self for problems inappropriately; unrealistically fears caretakers will be angry or rejecting, even after minor transgressions; excessively seeks reassurances that he or she is accepted as a good person, or makes other negative self-statements at a high frequency (and this is a change from baseline)  **Note**: People with Severe/Profound ID do not function at cognitive levels consistent with the capacity to experience or express feelings of guilt or worthlessness |
| 1. Diminished ability to think or concentrate, or indecisiveness, nearly every day (either by subjective account or as observed by others) | 8. No adaptation.  **Note:** observers may report the individual with ID: shows a reduced productivity at work or day program, has diminished self-care skills, appears easily distracted or can’t complete tasks he or she used to be able to finish, has shown the onset of or increase in agitated behaviors when asked to do activities that require concentration, has apparent memory problems that ”come and go”, has unexplained skill loss, shows an uncharacteristic inability to learn new skills as expected, or has had to stop working or attending programs due to poor performance |
| 1. Recurrent thoughts of death (not just fear of dying), recurrent suicidal ideation without a specific plan, or a suicide attempt or a specific plan for committing suicide | 9. No adaptation.  **Note:** observers may report the individual with Mild/Moderate ID: often talks about death or people who have died or has other morbid preoccupations, has frequent unrealistic or unfounded physical complaints and fears of illness or death, makes threats to kill or harm self or has actually attempted suicide (unconventional means such as running in front of cars or jumping from windows may be impulsive acts, but may be suicidal in nature) |
| B. The symptoms cause clinically significant distress or impairment in social, occupational, or other important areas of functioning | B. No adaptation.    **Note**: Individuals with ID may lose residential placements, jobs, vocational or other day programs due to apparent skill loss or associated disruptive behaviors, or there may be significant stress in the family or caretaking situation |
| C. The episode is not attributable to the physiological effects of a substance or to another medical condition  **Note**: Criteria A-C represent a major depressive episode    **Note**: Responses to a significant loss (e.g. bereavement, financial ruin, losses from a natural disaster, a serious medical illness or disability) may include the feelings of intense sadness, rumination about the loss, insomnia, poor appetite, and weight loss noted in Criterion A, which may resemble a depressive episode. Although such symptoms may be understandable or considered appropriate to the loss, the presence of a major depressive episode in addition to the normal response to a significant loss should also be carefully considered. This decision inevitably requires the exercise of clinical judgment based on the individual’s history and cultural norms for the expression of distress in the context of loss. | C. No adaptation.  **Note**: For people with ID, almost any physical problem that causes pain or distress may also cause difficulty focusing attention, sleeping, eating and psychomotor agitation. In addition to direct causes for mood problems (problems in thyroid functions), infections, common medical problems may provoke symptoms that look like depression. These medical problems include: UTIs, otitis media, cellulites, fungal infections of the skin., etc., constipation, GERD, migraine headaches, or a variety of medication induced movement disorders (akathisia, other EPS) or other drug side effects (lethargy, sedation, delirium). |
| D. The occurrence of the major depressive episode is not better explained by schizoaffective disorder, schizophrenia, schizophreniform disorder, delusional disorder, or other specified and unspecified schizophrenia spectrum and other psychotic disorders. | D. No adaptation |
| E. there has never been a manic episode or a hypomanic episode.  **Note**: This exclusion does not apply if all the manic-like or hypomanic-like episodes are substance-induced or are attributable to the physiological effects of another medical condition. | E. No adaptation |

Reprinted with permission from the Diagnostic and Statistical Manual of Mental Disorders, Fifth Edition, (Copyright 2013). American Psychiatric Association.

| **Schizophrenia** | | |
| --- | --- | --- |
| **DSM-5 Diagnostic Criteria** | **Apply Criteria for Mild and Moderate ID** | **Applying Criteria for Severe and Profound ID** |
| Please note: A significant change in behavior (for example, increased aggressive, self-injurious, or bizarre behavior) should alert the clinician to the possibility of a psychotic process. Assessment of this criterion might be especially difficult in individuals with severe or profound ID. | | |
| A. Two (or more) of the following, each present for a significant portion of time during a 1-month period (or less if successfully treated). At least one of these must (1), (2), or (3):  (1) Delusions.  (2) Hallucinations.  (3) Disorganized speech (e.g., frequent derailment or incoherence)  (4) Grossly disorganized or catatonic behavior.  (5) Negative symptoms (i.e., diminished emotional expression or avolition) | A. No adaptation | A. No adaptation  **Note**: There may be self-talk, which is common and not necessarily interpreted as an expression of psychotic disorder. |
| B. For a significant portion of the time since the onset of the disturbance, level of functioning in one or more areas, such as work, interpersonal relations, or self-care are markedly below the level achieved prior to the onset (or when the onset is in childhood or adolescence, there is failure to achieve expected level of interpersonal, academic or occupational functioning). | B. No adaptation | B. No adaptation |
| C. Continuous signs of the disturbance persist for at least 6 months. This 6-month period must include at least 1 month of symptoms (or less if successfully treated) that meet Criterion A (i.e., active-phase symptoms) and may include periods of prodromal or residual symptoms. During these prodromal or residual periods, the signs of the disturbance may be manifested by only negative symptoms or two or more symptoms listed in criterion A present in an attenuated form (e.g., odd beliefs, unusual perceptual experiences) | C. No adaptation | C. No adaptation |
| D. Schizoaffective disorder and depressive or bipolar disorder with psychotic features have been ruled out because either (1) no major depressive or manic episodes have occurred concurrently with the active-phase symptoms, or (2) if mood episodes have occurred during active-phase symptoms, they have been present for a minority of the total duration of the active and residual periods of the illness. | D. No adaptation | D. No adaptation |
| E. The disturbance is not attributable to the physiological effects of a substance (e.g., a drug of abuse, a medication) or another medical condition. | E. No adaptation | E. No adaptation |
| F. If there is a history of autistic spectrum disorder or a communication disorder of childhood-onset, the additional diagnosis of schizophrenia is made only if prominent delusions of hallucinations, in addition to the other required symptoms of schizophrenia, are also present for at least 1 month (or less if successfully treated). | F. No adaptation | F. No adaptation |
| Specify if  The following course specifiers are only to be used after a 1-year duration of the disorder and if they are not in contradiction to the diagnostic course criteria.  First episode, currently in acute episode: First manifestation of the disorder meeting the defining diagnostic symptom and time criteria. An acute episode is a time period in which the symptom criteria are fulfilled.  First episode, currently in partial remission: Partial remission is a period of time during which an improvement after a previous episode is maintained and in which the defining criteria of the disorder are only partially fulfilled.  First episode, currently in full remission: Full remission is a period of time after a previous episode during which no disorder-specific symptoms are present.  Multiple episodes, currently in acute episode: Multiple episodes may be determined after a minimum of two episodes (i.e., after a first episode, a remission and a minimum of one relapse).  Multiple episodes, currently in partial remission  Multiple episodes, currently in full remission  Continuous: Symptoms fulfilling the diagnostic symptom criteria of the disorder are remaining for the majority of the illness course, with subthreshold symptoms periods being very brief relative to the overall course.  Unspecified  Specify if:  With catatonia  Specific current severity:  Severity is rated by a quantitative assessment of the primary symptoms of psychosis, including delusions, hallucinations, disorganized speech, abnormal psychomotor behavior, and negative symptoms. Each of these symptoms may be rated for its current severity (most severe in the last 7 days) on a 5-point scale ranging from 0 (not present) to 4 (present and severe).  Note: Diagnosis of schizophrenia can be made without using this severity specifier |  |  |

Reprinted with permission from the Diagnostic and Statistical Manual of Mental Disorders, Fifth Edition, (Copyright 2013). American Psychiatric Association.

| **Schizoaffective Disorder** | | |
| --- | --- | --- |
| **DSM-5 Diagnostic Criteria** | **Applying Criteria for Mild and Moderate ID** | **Applying Criteria for Severe and Profound ID** |
| A. An uninterrupted period of illness during which there is a major mood episode (major depressive or manic) concurrent with Criterion A of Schizophrenia  Note: The Major Depressive Episode must include Criterion A1. | A. No adaptation | A. No adaptation * |
| B. Delusions or hallucinations for 2 or more weeks in the absence of a major mood episode (depressive or manic) during the lifetime duration of the illness. | B. No adaptation | B. No adaptation * |
| C. Symptoms that meet criteria for a major mood episode are present for the majority of the total duration of the active and residual portions of the illness. | C. No adaptation | C. No adaptation * |
| D. The disturbance is not attributable to the effects of a substance or another medical condition. | D. No adaptation | D. No adaptation * |
| Specify whether:  Bipolar type: This subtype applies if a manic episode is part of the presentation. Major depressive episodes may also occur.  Depressive type: This subtype applies if only major depressive episodes are part of the presentation.  Specify if: With catatonia  Specify if: The following course specifiers are only to be used after a 1-year duration of the disorder and if they are not in contradiction to the diagnostic course criteria.  First episode, currently in acute episode: First manifestation of the disorder meeting the defining diagnostic symptom and time criteria. An acute episode is a time period in which the symptom criteria are fulfilled.  First episode, currently in partial remission: Partial remission is a period of time during which an improvement after a previous episode is maintained and in which the defining criteria of the disorder are only partially fulfilled.  First episode, currently in full remission: Full remission is a period of time after a previous episode during which no disorder-specific symptoms are present.  Multiple episodes, currently in acute episode: Multiple episodes may be determined after a minimum of two episodes (i.e., after a first episode, a remission and a minimum of one relapse).  Multiple episodes, currently in partial remission  Multiple episodes, currently in full remission  Continuous: Symptoms fulfilling the diagnostic symptom criteria of the disorder are remaining for the majority of the illness course, with subthreshold symptoms periods being very brief relative to the overall course.  Unspecified  Specific current severity:  Severity is rated by a quantitative assessment of the primary symptoms of psychosis, including delusions, hallucinations, disorganized speech, abnormal psychomotor behavior, and negative symptoms. Each of these symptoms may be rated for its current severity (most severe in the last 7 days) on a 5-point scale ranging from 0 (not present) to 4 (present and severe).  Note: Diagnosis of schizoaffective disorder can be made without using this severity specifier |  |  |

* The criteria and adaptations for schizophreniform disorder are identical to those for schizophrenia, with the exception that criteria B on social/occupation dysfunction is not required; and that the duration criteria is for at least 1 month but less than 6 months. When the diagnosis is made without waiting for recovery, it should be qualified as “provisional”. Reprinted with permission from the Diagnostic and Statistical Manual of Mental Disorders, Fifth Edition, (Copyright 2013). American Psychiatric Association.

| **Catatonia Associated with Another Medical Disorder (Catatonia Specifier)** | | |
| --- | --- | --- |
| **DSM-5 Diagnostic Criteria** | **Applying Criteria for Mild and Moderate ID** | **Applying Criteria for Severe and Profound ID** |
| A. The clinical picture is dominated by three (or more) of the following symptoms:   1. Stupor (i.e., no psychomotor activity; not actively relating to environment). 2. Catalepsy (i.e., passive induction of a posture held against gravity). 3. Wavy flexibility (i.e., slight, even resistance to positioning by examiner). 4. Mutism (i.e., no, or very little, verbal response [(exclude if known aphasia]). 5. Negativism (i.e., opposition or no response to instructions or external stimuli).   Posturing (i.e., spontaneous and active maintenance of a posture against gravity). Mannerism (i.e., odd, circumstantial caricature of normal actions). Stereotypy (i.e., repetitive, abnormally frequent, non-goal-directed movements). Agitation, not influenced by external stimuli. Grimacing. Echolalia (i.e., mimicking another’s speech). Echopraxia (i.e., mimicking another’s movements).  Coding note: Indicate the name of the associated mental disorder when recording the name of the condition (catatonia associated with major depressive disorder). Code first the associated mental disorder (e.g., neurodevelopmental disorder, brief psychotic disorder, schizophreniform disorder, schizophrenia, schizoaffective disorder, bipolar disorder, major depressive disorder, or other mental disorder). | A. No adaptation | **Note**: Mutism, mannerisms, stereotypies, and grimacing can be features of ID, and echolalia can be a feature of autism spectrum disorder. A history of time of onset of these symptoms may helpfully differentiate. |

Reprinted with permission from the Diagnostic and Statistical Manual of Mental Disorders, Fifth Edition, (Copyright 2013). American Psychiatric Association.

| **Obsessive-Compulsive Disorder** | | |
| --- | --- | --- |
| **DSM-5 Diagnostic Criteria** | **Applying Criteria for Mild to Moderate ID** | **Applying Criteria for Severe to Profound ID** |
| A. Presence of obsessions, compulsions, or both | A. No adaptation | A. No adaptation |
| Obsessions are defined by:   1. Recurrent and persistent thoughts, urges, or images that are experienced, at some time during the disturbance, as intrusive and unwanted, and that in most individuals cause marked anxiety or distress. 2. The individual attempts to ignore or suppress such thoughts, urges, or images, or to neutralize them with some other thought or action (i.e., by performing a compulsion). | Obsessions are defined by (1) and (2)   1. No adaptation   **Note**: Recurrent and persistent thoughts, urges, or images may not be experienced as intrusive and unwanted depending on the cognitive functioning of the individual.   1. No adaptation   **Note**: The individual may or may not (due to cognitive deficits) attempt to ignore or suppress such thoughts, urges, or images, or to neutralize them with some other thought or action. | Obsessions are defined by (1) and (2)   1. No adaptation   **Note**: Recurrent and persistent thoughts, urges, or images may not be experienced as intrusive and unwanted depending on the cognitive functioning of the individual.   1. No adaptation   **Note**: The individual may or may not (due to cognitive deficits) attempt to ignore or suppress such thoughts, urges, or images, or to neutralize them with some other thought or action. The individual may be unable to report wanting to ignore, suppress, or neutralize the obsessions. |
| Compulsions are defined by (1) and (2):   1. Repetitive behaviors (e.g., hand washing, ordering, checking) or mental acts (e.g., praying, counting, repeating words silently) that the individual feels driven to perform in response to an obsession or according to rules that must be applied rigidly. 2. The behaviors or mental acts are aimed at preventing or reducing anxiety or distress, or preventing some dreaded event or situation; however, these behaviors or mental acts are not connected in a realistic way with what they are designed to neutralize or prevent, or are clearly excessive. Note: Young children may not be able to articulate the aims of these behaviors or mental acts. | Compulsions are defined by (1) and (2):   1. No adaptation   **Note**: Repetitive behaviors or mental acts may be difficult to elicit due to cognitive deficits and limited expressive language skills. Consider ordering, telling, asking, or repetitive physical acts (e.g., rubbing) as compulsions.   1. No adaptation   **Note**: The function of the compulsive behavior may not be ascertainable due to cognitive deficits and limited expressive language sills, recognition of excessiveness or intent of the behaviors may not be present. | Compulsions are defined by (1) and (2):   1. No adaptation   **Note**: Absence of compulsions that require abstract thinking does not rule out OCD; observe individuals for compulsions requiring simple thinking, such as fixed sequences or arrangements, excessive ordering, and filling/emptying.     1. No adaptation   **Note**: The function of the compulsive behavior may not be ascertainable due to cognitive deficits and limited expressive language skills. The criteria regarding intent of the behavior does not apply to children, and does not apply to individuals with severe/profound intellectual disability. |
| B. The obsessions or compulsions are time-consuming (e.g., take more than 1 hour per day) or cause clinically significant distress or impairment in social, occupational, or other important areas of functioning. | B. No adaptation  **Note**: Distress may not occur and/or may not be ascertainable. Intense preoccupation may be observed or drive to perform the compulsion may be observed. Challenging behavior, especially aggression, and self-injurious behavior, may occur if the individual is prevented from completing the compulsion. | B. No adaptation  **Note**: Distress may not occur and/or may not be ascertainable. Intense preoccupation, strong urges to engage in compulsive activity may be observed. Aggression, especially directed toward caregivers who impede the completion of the compulsion, may be seen. |
| Specify if:  Good Insight, Fair Insight, Poor Insight or Absent/Delusional Beliefs | The various specifiers rating patient’s insight into disorder-related beliefs (e.g. good, fair, poor or absent/delusional beliefs) should be applied in the context of the cognitive and developmental functioning of the individual. | The various specifiers rating patient’s insight into disorder-related beliefs (e.g. good, fair, poor or absent/delusional beliefs) should be applied in the context of the cognitive and developmental functioning of the individual. |
| C. The obsessive-compulsive symptoms are not attributable to the physiological effects of a substance (e.g., a drug of abuse, a medication) or another medical condition. | C. The various specifiers rating patient’s insight into disorder-related beliefs (e.g. good, fair, poor or absent/delusional beliefs) should be applied in the context of the cognitive and developmental functioning of the individual. | C. The various specifiers rating patient’s insight into disorder-related beliefs (e.g. good, fair, poor or absent/delusional beliefs) should be applied in the context of the cognitive and developmental functioning of the individual. |

Reprinted with permission from the Diagnostic and Statistical Manual of Mental Disorders, Fifth Edition, (Copyright 2013). American Psychiatric Association.
